# From Triplet to Twist: The Photochemical *E*/*Z*‐Isomerization Pathway of the Near‐Infrared Photoswitch *peri*‐Anthracenethioindigo

**DOI:** 10.1002/anie.202510626

**Published:** 2025-08-07

**Authors:** Martina Hartinger, Maximilian Herm, Christoph Schüßlbauer, Laura Köttner, Dirk Guldi, Henry Dube, Carolin Müller

**Affiliations:** ^1^ Friedrich‐Alexander‐Universität Erlangen‐Nürnberg Computer Chemistry Center Nägelsbachstraße 25 91052 Erlangen Germany; ^2^ Friedrich‐Alexander‐Universität Erlangen‐Nürnberg Egerlandstraße 3 91054 Erlangen Germany; ^3^ Friedrich‐Alexander‐Universität Erlangen‐Nürnberg Nikolaus‐Fiebiger Straße 10 91054 Erlangen Germany

**Keywords:** Excited state theory, Near infrared photoswitching, *peri*–anthracenethioindigo, Photoswitch mechanism, Ultrafast spectroscopy

## Abstract

In recent years, large progress has been made to shift the absorption of photoresponsive molecules into the long‐wavelength region of the electromagnetic spectrum. A breakthrough in this field was the recent development of π‐extended indigoid photoswitches, i.e., *peri*‐anthracenethioindigo (PAT), exhibiting all‐red and near‐infrared addressability. The excited‐state isomerization mechanisms of this very new addition to the realm of photoswitching are currently not understood at all, prohibiting a rational further development. In this study, we present a combined theoretical and experimental approach, including time‐dependent density functional theory (TD‐DFT) and second‐order algebraic diagrammatic construction (ADC(2)) calculations as well as steady‐state and time‐resolved femtosecond spectroscopy, to explore the isomerization pathways of this photoswitch. Our findings show that photoisomerization on singlet potential energy surfaces (PESs) is highly unfavorable and instead show that photoswitching proceeds on the $T_{1}$ PES. These insights enable a deep understanding of thioindigoid photochemistry and demonstrate that extension of the π‐system and *peri*‐connectivity in the heterocycle unlock extremely favorable photoswitching properties along with the desirable red‐shift in absorption. Reliable photoswitching from the triplet is achieved because of its favorable energy, which evades undesirable interference of oxygen quenching. These results pave the way for advancing thioindigoid‐based photoswitches to improved performance and functionality in a rational way.

## Graphical abstract

The full photoisomerization mechanism of the all‐red‐light addressable *peri*‐anthracenethioindigo (PAT) has been decoded. Theory and transient absorption spectroscopy show that both E→Z and Z→E switching proceed via the triplet state. Competing pathways differ: whereas E‐PAT shows a flat $T_{1}$ minimum, Z‐PAT undergoes fast singlet deactivation. These insights explain PAT's efficient bidirectional switching and guide rational design.

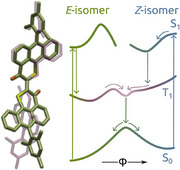

## Introduction

In the realm of photochemistry, molecular structures with the ability to undergo reversible changes in structural and electronic properties upon exposure to light have captivated the scientific community. Among them, photoswitches and molecular motors stand out as versatile agents, capable of transforming light energy into directed mechanical motion, with potential applications across a broad spectrum, including molecular machines,^[^ ^1^ ^]^ catalysis,^[^ ^2^, ^3^, ^4^ ^]^ materials sciences,^[^ ^5^, ^6^, ^7^, ^8^ ^]^ and chemical biology.^[^ ^9^, ^10^, ^11^, ^12^ ^]^ However, one of the greatest remaining challenges hindering general applicability, especially in the context of biology, is the reliance on higher‐energy light, mostly UV or blue light, to drive these processes. This restriction is severely limiting, as high‐energy irradiation leads to undesirable photodestructive effects, inhibition of selective control in multi‐responsive systems, and insufficient penetration depth into biological tissues, as well as cellular damage. As a result, recent years have seen an increased research focus on the development of red‐light responsive systems^[^ ^13^ ^]^ (see Ref. [14] for a review). For example, substituted and bridged azobenzenes,^[^ ^15^ ^]^ substituted diarylethenes,^[^ ^16^, ^17^ ^]^ substituted indigo,^[^ ^18^, ^19^, ^20^ ^]^ hemiindigo,^[^ ^21^, ^22^, ^23^ ^]^ and indirubin^[^ ^24^ ^]^ were introduced, with at least one of their isomers being able to absorb red light. Despite this progress, most of the studied systems rely on thermal backreactions or require high‐energy visible light for the reverse photoreaction. Moreover, short thermal half‐lives of metastable isomers inhibit true photocontrol in many switches.

**Figure 1:**
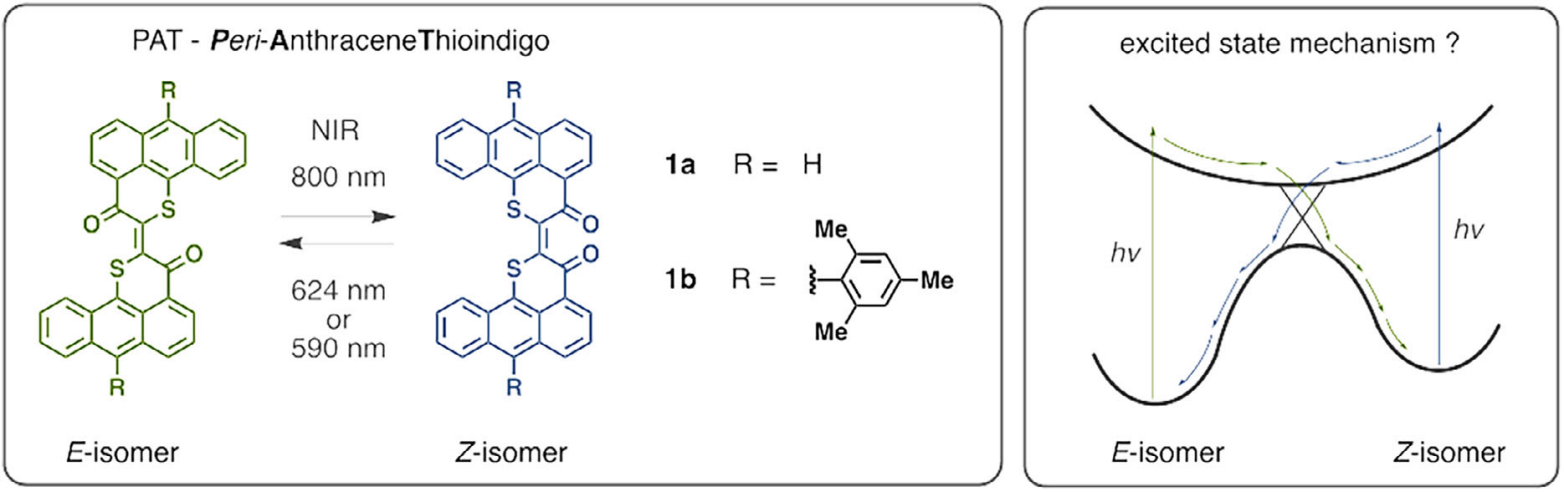
Photoisomerization of PAT photoswitches **1a** and **1b**. Continuous irradiation conditions established all‐red and NIR light responsiveness,^[^ ^25^ ^]^ enabling direct excitation of the *E*‐isomer with 800 nm light and of the *Z*‐isomer with 590 nm light. The excited state mechanism was completely unknown at the outset of this study.

Very recently, *peri*‐anthracenethioindigo (PAT)^[^ ^25^ ^]^ was introduced as an all‐red‐light‐responsive photoswitch (Figure 1). The PAT photoswitch is related to *peri*‐thioindigoid photoswitches, especially the naphthalene derivatives, which were first synthesized by Friedländer in 1912^[^ ^26^ ^]^ and investigated by Oksengendler and Mostoslavskii in 1959^[^ ^27^ ^]^ and the 1960s.^[^ ^28^, ^29^ ^]^ Due to the extended π‐system in PAT, both *E*‐ and *Z*‐isomers absorb in the red‐light to near infrared (NIR) part of the electromagnetic spectrum, marking a milestone in the development of low‐energy responsive molecular photoswitches. Outstanding photochemical and photophysical properties of the PAT photoswitch were obtained, including large negative photochromism, substantial quantum yields, and high thermal stability of the metastable *Z*‐isomer, leading to days long persistence of this state. This enabled the first truly orthogonal photoswitching system that is fully path‐independent and can be operated exclusively with only visible light signaling.^[^ ^30^ ^]^ For any further progress in the development and application of such extremely low‐energy light responsive photoswitching, a detailed understanding of the fundamental mechanisms is of paramount importance. Without it, no rational design and informed tailoring of properties can be achieved. Although thioindigoid dyes have been the subject of photophysical and mechanistic investigations since the 1970s,^[^ ^31^, ^32^, ^33^, ^34^, ^35^, ^36^ ^]^ no such study is currently available for the all‐red‐ and NIR‐responsive PAT photoswitch. Because of the particularly low‐energy light able to excite PAT, it could be suspected that its excited‐state potential surface, and hence photoisomerization mechanism differ substantially from its visible‐light‐responsive thioindigoid relatives. Earlier studies on thioindigoids suggested that the photoisomerization mechanism involves different types of electronic states for the *E*‐to‐*Z* and *Z*‐to‐*E* directions. While for the *E*‐to‐*Z* direction a triplet pathway is evident,^[^ ^31^, ^32^, ^33^ ^]^ the *Z*‐to‐*E* direction shows a more complex behavior involving both singlet and triplet states in the photoinduced processes.^[^ ^34^, ^35^, ^36^, ^37^ ^]^ In contrast, the outstanding air stability of PAT photoswitching observed under continuous irradiation conditions does not readily hint at a triplet mechanism from the outset.^[^ ^25^ ^]^

In this work, we explore the photoisomerization mechanism of the PAT photoswitch using ultrafast transient absorption spectroscopy (**1b**) and quantum chemical calculations (**1a** and **1b**, see Figure 1). Together these methods provide comprehensive insights into the nature and dynamics of the PAT excited states from which a competent future rational design of this emerging class of low‐energy light‐responsive photoswitches can be build.

## Results and Discussion

To elucidate the photoisomerization behavior of **1b**, we adopted a two‐part strategy, structured into static and dynamic approaches. Such an approach has proven very successful when describing the photochemical mechanisms of indigoids,^[^ ^38^, ^39^, ^40^, ^41^ ^]^ in particular for hemithioindigo.^[^ ^42^, ^43^, ^44^, ^45^, ^46^, ^47^, ^48^, ^49^, ^50^ ^]^

First, we examine static properties, which are based on quantum chemical calculations, and reveal the potential energy landscape along a proposed isomerization coordinate. This theoretical analysis provides mechanistic insight into the structural and energetic changes accompanying the photoisomerization. The subsequent dynamic section builds on these findings by investigating the excited‐state behavior using femtosecond and nanosecond transient absorption spectroscopy. Simulated transient absorption spectra at key geometries along the computed reaction pathway serve to guide the interpretation of the experimental data.

### Static Properties

The static part of the discussion focuses on the fundamental photophysical properties of the *E*‐ and *Z*‐isomers of **1b**. We first analyze the ground state absorption properties to understand the properties in the Franck–Condon region. This is followed by a computational exploration of a hypothetical reaction coordinate connecting the two isomers. This theory‐proposed static coordinate is allowing us to establish the energetic landscape associated with the isomerization pathway.

#### Franck–Condon Region

**Figure 2:**
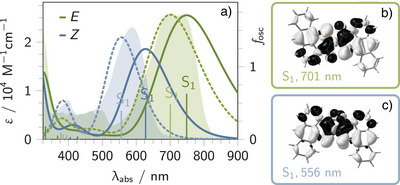
Absorption spectra of the *E*‐ and *Z*‐isomers of **1a** and **1b**. Experimental absorption spectra (a, shaded curves) of **1b**‐*E* (green) and **1b**‐*Z* (blue) in tetrahydrofuran, along with simulated spectra of **1a**‐*E*/*Z* (a, non‐shaded curves). Simulated spectra were obtained using TD‐DFT (B3LYP/6‐311G(d,p), solid lines) and ADC(2)/def2‐TZVP (a, dashed lines) both considering implicit solvent effects (ε=2.2706). Vertical excitation energies and oscillator strengths are indicated as bars for both isomers (green or blue), with dark and light shades representing the two computational methods. Spectra were generated by Gaussian broadening (full width at half maximum of 0.28 eV). Charge density differences from TD‐DFT simulations of the bright $S_{0}\to S_{1}$ transition for **1a**‐*E* b) and **1a**‐*Z* c) show electron density shifting from light gray to black regions upon excitation.

To investigate the effect of light excitation on electronic transitions within the Franck–Condon region, we studied the *E*‐ and *Z*‐isomers of **1b** using steady‐state absorption spectroscopy supported by quantum chemical calculations. The corresponding experimental and simulated absorption spectra are shown in Figure 2a and Figure S1.

The ground‐state absorption spectra of **1b** exhibit three characteristic features: a strong absorption near 260 nm and weaker ones between 350 and 540 nm, both assigned to localized $\pi \pi^{*}$ transitions that are anthracene centered. In contrast, the broad, intense low‐energy absorptions with maxima at 550 nm (**1b**‐*Z*) and 725 nm (**1b**‐*E*)^[^ ^25^ ^]^ are attributed to charge‐transfer transitions (see filled curves in Figure 2a). This is supported by time‐dependent density functional theory (TD‐DFT) simulations, which predict a bright first singlet excited state ($S_{1}$) with pronounced charge‐transfer character for **1b**‐*E* (770 nm) and **1b**‐*Z* (636 nm, see B3LYP results in Table S1). In this charge‐transfer excitation, electron density is shifted from the sulfur atoms, the anthracenes, and the central double bond mainly to the thioindigo and carbonyl fragments, leading to charge localization on the central photoswitching unit. Of particular interest is that the central isomerizable double bond is broken upon this excitation. Higher singlet excited states ($S_{2,3,4}$) exhibit low oscillator strengths and contribute only marginally to the low‐energy absorptions (cf. Tables S2 and S3).

Since the mesityl groups in **1b** are not involved in the key charge‐transfer transitions, we employed a simplified model, **1a**, in which the mesityl substituents are replaced by a hydrogen atom, to reduce computational costs without compromising qualitative accuracy. The similar electronic behavior of **1a** and **1b** is reflected in their absorption features as obtained by means of TD‐DFT (see Tables S1–S3). Furthermore, the simulated spectra of **1a** align well with the experimental data for **1b** (compare unfilled vs. filled curves in Figure 2a and Figure S2), confirming the reliability of our computational model.

For both **1a** and **1b**, TD‐DFT systematically underestimates the vertical excitation energy of the low‐energy absorption.^[^ ^25^ ^]^ A comparison of the TD‐B3LYP results for **1a** with those obtained using second‐order algebraic diagrammatic construction, ADC(2), shows that ADC(2) slightly overestimates the excitation energies but provides a closer match to the experimental spectra (see dashed lines vs. filled curves in Figure 2a). Although the absolute oscillator strengths differ between methods, the relative intensity ratio of the *E*‐ and *Z*‐isomers (TD‐B3LYP: 1.4, ADC(2): 1.3) is in good agreement with the experimental ratio of 1.1.

#### *E*/*Z*‐Isomerization Potential Energy Curves

A common theoretical approach to describe *E*/*Z*‐isomerization involves the static sampling of geometries along a reaction coordinate described by torsion angles around the central double bond. For many switches, like simple alkenes, cyanine dyes, or azobenzene dyes, the potential energy surface (PES) of $S_{1}$ typically intersects with that of the ground state ($S_{0}$) near a dihedral angle of 90.^[^ ^51^, ^52^ ^]^ This conical intersection enables relaxation to $S_{0}$, allowing to either complete the isomerization or return to its original configuration.

**Figure 3:**
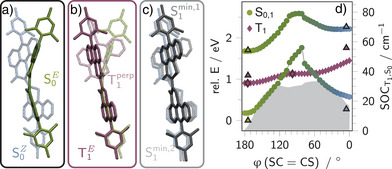
Stationary structures and energy profile along the minimum energy path connecting **1b**‐*E* and **1b**‐*Z* on the $T_{1}$ surface. Visualization of the equilibrium geometries of **1b**‐*E* (green, $S_{0}^{E}$) and ‐*Z* (blue, $S_{0}^{Z}$) in the ground state a), and local minimum geometries in the $T_{1}$ (b, $T_{1}^{E}$ and $T_{1}^{\mathrm{perp}}$) and $S_{1}$ (c, $S_{1}^{\min,1}$ and $S_{1}^{\min,2}$). For clarity, the hydrogen atoms were omitted in the structure representations. d) Potential energy curves for $S_{0}$ and $S_{1}$ (circles) and $T_{1}$ (diamonds) obtained via constrained geometry optimizations on the $T_{1}$ surface at torsion angles ranging from 180 (**1b**‐*E*) to 0 (**1b**‐*Z*). The respective spin‐orbit couplings (SOCs) between $S_{0}$ and $T_{1}$ are shown as a gray filled curve. The energies of minimum geometries are indicated by symbols with black outlines. The Franck–Condon point energies (geometries shown in a) are indicated by triangles. All geometries, energies and couplings were computed at (TD‐)DFT/6‐311G(d,p) level of theory.

To model this process for **1b**, a relaxed scan of the central SC = CS torsional angle was performed on the triplet excited state ($T_{1}$) surface, ranging from 180 (*E*‐configuration) to 0 (*Z*‐configuration). The corresponding energies in the singlet manifold ($S_{0,1,2}$) and triplet manifold ($T_{2}$) were subsequently calculated at each of these optimized geometries (see Figure 3 and Figure S2).

The actual structural rearrangement during double bond isomerization is more complex, since the *E*‐isomer exhibits a bowl‐shaped geometry, while the *Z*‐isomer adopts a stepped conformation^[^ ^25^ ^]^ across the conjugated backbone (see simplified geometries in Figure 3a or and overlay of geometries in Figure S4). The initial flattening of the bowl shape, the development of the step‐like distortion, and the rotation around the dihedral angle are highly unlikely to occur along a single reaction coordinate. Therefore, we show the relative energies (relative to $S_{0}^{E}$) of the simplified torsional scan. The relative energies of the equilibrium geometries of $S_{0}^{E}$, $S_{0}^{Z}$, and $T_{1}^{\mathrm{perp}}$ are illustrated by the black‐outlined symbols. Noteworthy, the ground state geometries do not coincide with the $T_{1}$‐relaxed geometries (see e.g., $T_{1}^{E}$ vs. $S_{0}^{E}$ in Figure S4) and are just illustrated for comparison.

Along this pathway, when starting from the *E*‐isomer, the electronic energies of both $S_{0}$ and $S_{1}$ increase steadily as the system approaches a perpendicular geometry, reaching their respective maxima near a 90° dihedral angle. Specifically, $S_{0}$ and $S_{1}$ rise by 1.6 and 0.9 eV, respectively (cf. circle symbols in Figure 3). Higher singlet excited states ($S_{2}$, $S_{3}$) show similar potential energy profiles, making the perpendicular geometry energetically inaccessible within the singlet manifold along this specific path (see Figure S3).

In contrast, the potential energy curve of $T_{1}$ remains relatively flat at the same coordinate, indicating a potentially more favorable route for photoisomerization through the triplet manifold. This potential energy surface reveals two local minima (see light‐purple vs. purple geometry in Figure 3b): one corresponding to a fully planar *E*‐configured geometry ($T_{1}^{E}$), and another, 4.0 kcal ${\mathrm{mol}}^{-1}$ higher in energy, with a perpendicular arrangement of the two fragments ($T_{1}^{\mathrm{perp}}$, *cf*. purple highlighted geometries in Figure 3b). These minima are separated by a transition state featuring an energy barrier of 5.6 kcal ${\mathrm{mol}}^{-1}$. Such a double‐minimum topology agrees with our findings for the thioindigo core motif (see Figure S3) and previous studies on thioindigoid systems with similar geometries.^[^ ^34^ ^]^

Further analysis of excited‐state coupling at the *E*‐configuration reveals that $S_{1}$ (1.6 eV) and $T_{2}$ (1.4 eV) are nearly degenerate at the Franck–Condon geometry ($S_{0}^{E}$). This suggests that a population transfer from $S_{1}$ to $T_{2}$ is more likely than to $T_{1}$ (1.0 eV). Indeed, the calculated spin‐orbit coupling (SOC) values support this, showing an $S_{1}$/$T_{2}$ coupling of 2.14 ${\mathrm{cm}}^{-1}$ versus 0.06 ${\mathrm{cm}}^{-1}$ for $S_{1}$/$T_{1}$ (see also Table S8). At the planar $T_{1}$‐minimum, all SOCs are negligible (≤0.01 ${\mathrm{cm}}^{-1}$) and intersystem crossing (ISC) is inefficient. The perpendicular $T_{1}$ minimum shows a much larger SOC with the ground state (see gray shaded curve in Figure 3d), e.g., 30.47 ${\mathrm{cm}}^{-1}$ at the crossing point. In turn, reverse ISC ($T_{1}\to S_{0}$) can occur rapidly at geometries with dihedrals ranging from around 130 to 50 (see Figure 3d).

For the *Z*‐isomer of PAT, two minima were located on the $S_{1}$ surface with SC = CS dihedral angles of 16 ($S_{1}^{\min,1}$) and 37 ($S_{1}^{\min,2}$), respectively. The latter represents the energetically favored geometry, which is stabilized by 0.42 eV (see also Figure S4). Both structures are flattened and twisted compared to the step‐like ground‐state conformation, which shows a dihedral angle of 5, reflecting significant structural reorganization upon excitation. These geometries are illustrated in Figure 3c.

Like for the *E*‐isomer, SOC values between $S_{1}$ and $T_{2}$ are larger ($S_{1}^{\min,1}$: 11.96 ${\mathrm{cm}}^{-1}$; $S_{1}^{\min,2}$: 4.89 ${\mathrm{cm}}^{-1}$) than those with $T_{1}$ ($S_{1}^{\min,1}$: 2.62 ${\mathrm{cm}}^{-1}$; $S_{1}^{\min,2}$: 3.02 ${\mathrm{cm}}^{-1}$), though all SOCs are higher than in the corresponding *E*‐isomer (see also Table S8). Importantly, the $T_{1}$/$S_{0}$ SOC at the $S_{1}^{\min,2}$ geometry (31.23 ${\mathrm{cm}}^{-1}$) is comparable to that of the perpendicular $T_{1}$ minimum ($\varphi =93^{\circ }$, 30.14 ${\mathrm{cm}}^{-1}$), indicating that a non‐productive deactivation to the ground state may occur already at moderately twisted geometries such as 37, before the system reaches the fully twisted configuration required for a productive photoisomerization.

### Photoinduced Dynamics

To elucidate the time‐resolved aspects of photoisomerization and to obtain direct experimental evidence for the theoretically predicted behavior, we investigated these processes using transient absorption spectroscopy. The ultrafast processes were monitored by means of femtosecond spectroscopy. To observe the full excited state decay, nanosecond transient absorption experiments were performed.

**Figure 4:**
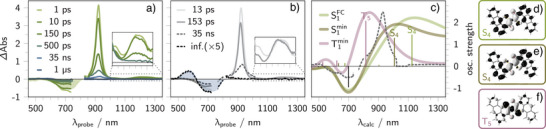
Experimental and simulated transient absorption spectra for *E*‐to‐*Z* photoisomerization. a) Transient absorption spectra of **1b**‐*E* upon 750 nm excitation in argon‐purged THF at room temperature. The filled curve shows the scaled and inverted ground‐state absorption spectrum of **1b**‐*E*. b) Evolution‐associated spectra (EAS) extracted from global analysis. The blue filled curve represents the difference between the ground‐state absorption spectra of **1b**‐*Z* and **1b**‐*E*. c) Simulated transient absorption difference spectra (solid lines) for excitations from the **1b**‐*E* ground state ($S_{0}$) to $S_{1}^{\mathrm{FC}}$ (light green), $S_{1}^{E,\min}$ (dark green), and $T_{1}^{E,\min}$ (purple). For comparison, the experimental EAS associated with the 35 ns time constant is shown as a dashed line. Vertical excitation energies and oscillator strengths are indicated as bars (negative for $S_{0}$, positive for excited states) and were spectrally broadened using Gaussian functions (full‐width at half‐height of 0.28 eV). d–f) Charge density differences of the main ESA transition in $S_{1}^{\mathrm{FC}}$, $S_{1}^{E,\min}$, and $T_{1}^{E,\min}$ configurations.

**Figure 5:**
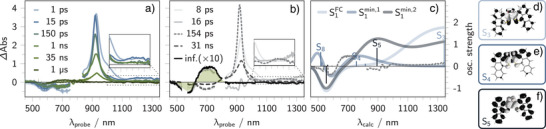
Experimental and simulated transient absorption spectra for *Z*‐to‐*E* photoisomerization. a) Transient absorption spectra of **1b**‐*Z* upon 550 nm excitation in argon‐purged THF at room temperature. The filled curve shows the scaled and inverted ground‐state absorption spectrum of **1b**‐*Z*. b) Evolution‐associated spectra (EAS) extracted from global analysis. The green filled curve represents the difference between the ground‐state absorption spectra of **1b**‐*E* and **1b**‐*Z*. c) Simulated transient absorption difference spectra (solid, opaque lines) for vertical excitations from the **1b**‐*Z* ground state ($S_{0}$) to the $S_{1}$ state considering three different geometries: the Franck–Condon geometry ($S_{1}^{\mathrm{FC}}$, light blue), a minimum with flattened chromophore ($S_{1}^{\min,1}$, blue), and a flattened, twisted geometry ($S_{1}^{\min,2}$, dark blue). For comparison, the experimental EAS associated with the 16 ps time constant is shown as a dashed line. Vertical excitation energies and oscillator strengths are indicated as bars (negative: $S_{0}\to S_{n}$, positive: $S_{1}\to S_{n+1}$) and were spectrally broadened using Gaussian functions (FWHM = 0.3 eV). d–f) Charge density differences (excitation from white to black) of a major ESA feature in $S_{1}^{\mathrm{FC}}$ d), $S_{1}^{\min,1}$ e), and $S_{1}^{\min,2}$ f).

To support the interpretation, the experimental data are complemented by simulated transient absorption spectra along the isomerization coordinate (see Figures 4c and 5c, and Figure S8). Given that **1b**‐*E* is thermodynamically more stable than **1b**‐*Z*, our analysis starts with the *E*‐to‐*Z* isomerization. The reverse process, light‐induced *Z*‐to‐*E* isomerization, requires prior photoconversion of the *E*‐isomer to the *Z*‐form and is addressed in the final part of this section.

#### *E*‐to‐*Z* Photoisomerization

Upon 750 nm photoexcitation, femtosecond transient absorption (TA) spectra of **1b**‐*E* in tetrahydrofuran show an immediate ground state bleaching (GSB) between 600 and 750 nm, reflecting the diminished ground state absorption of **1b**‐*E* (see Figure 4a). This GSB is accompanied by a strong excited state absorption (ESA) centered at 922 nm and weaker ESA features in the 450–550 nm and 1100–1300 nm ranges, and an ESA shoulder at 1050 nm. Within the first 13 ps, the ESA shoulder at around 1050 nm vanishes and the 922 nm ESA maximum decreased in intensity. Within the next 153 ps, this ESA maximum is subject to a red‐shift from 922 to 930 nm and the ESA between 1100 and 1300 nm disappears. It is within 35 ns that the 930 nm ESA maximum is replaced by features in the positive signal region between 500 and 650 nm accompanied by GSB between 650 and 750 nm (cf. black line in Figure 4b). These spectral signatures match the ground‐state absorption of **1b**‐*Z*, indicating successful photoisomerization and the formation of the *Z*‐isomer as a stable photoproduct.

Global lifetime analysis identified three characteristic time constants: 13 ps, 153 ps, and 35 ns (see Figure 4b). The spectral changes associated with the first characteristic time constant, describe the decrease of ESAs between 800 and 1100 nm causing a sharpening and intensity decrease of the ESA at 922 nm. The distinct features at 922 and 1050 nm are attributed to charge transfer transitions, where electron density is shifted from the central thioindigo moiety toward the anthracene moieties ($S_{1}\to S_{4,5}$, see charge density difference in Figure 4d and Tables S4). We associate the decrease of these signals with vibrational cooling of the initially populated hot singlet excited state ($S_{1}^{\mathrm{hot}}$) whereby $S_{1}$ is formed. TD‐DFT calculations indicate that this $S_{1}$ minimum adopts a similar bowl‐shaped structure like the **1b**‐*E* ground state minimum geometry (see $S_{0}^{E}$ vs. $S_{1}^{E}$ in Figure S3). According to our ADC(2) simulations, the respective $S_{1}^{E,\min}$ state shows a strong ESA at 1007 nm ($S_{1}\to S_{4}$, see charge density difference in Figure 4e), which appear blue‐shifted with respect to the main ESA of the Franck–Condon geometry ($S_{1}\to S_{4}$: 1124 nm, cf. Figure 4c).

The so‐formed $S_{1}$ undergoes ISC to $T_{1}$, likely via $T_{2}$, as suggested by the higher $S_{1}$/$T_{2}$ spin‐orbit couplings compared to $S_{1}$/$T_{1}$ in the *E*‐geometries (see Table S8), with the second characteristic time constant of 153 ps, where the rather fast ISC stems from the heavy‐atom effect introduced by the sulfur atoms. This is reflected in the emergence of a new ESA around 930 nm, which is red‐shifted relative to the original 922 nm ESA and the simultaneous decay of ESAs ranging from 1100 to 1300 nm. Independent support came from theory, showing no relevant triplet–triplet ESA features at wavelengths beyond 1000 nm, whereas singlet–singlet ESA features are found in this region ($S_{1}\to S_{1-5}$, see also Tables S6). Instead, the quantum chemical simulations reveal that $T_{1}$ shows a dominant absorption at 815 nm ($T_{1}\to T_{5}$) or 960 nm ($T_{1}\to T_{6}$) at the ADC(2) (see Figure 4c) and TD‐DFT levels of theory, respectively (see Figure S8). This coincides with the experimentally observed species‐associated spectrum, showing an ESA maximum at around 940 nm (compare the dashed and purple line in Figure 4c). According to both levels of theory, this ESA characteristic is associated with a $\pi \pi^{*}$ transition localized on the thioindigo core motif (see the charge density differences in Figures 4f and Figure S4).

The third characteristic time constant (35 ns) describes the build‐up of signals in the positive signal region between 500 and 650 nm at the expense of the ESA associated with $T_{1}$ between 800 and 1000 nm. As the spectral shape of the features between 500 and 650 nm reflects the shape of the ground state absorption spectrum of **1b**‐*Z* and the fact that these signals remain as infinite components (infinite lifetime), we associate this process with the *E*‐to‐*Z* isomerization of **1b** in the triplet manifold. This is supported by a differential spectrum formed between the ground state absorption spectra of **1b**‐*E* and **1b**‐*Z* (see the black dotted line in Figure 4b).

The kinetics of this process are rationalized by the computed triplet potential energy surface. The planar *E*‐isomer $T_{1}$ geometry, which is initially populated from the $S_{1}$ state, is 4 kcal ${\mathrm{mol}}^{-1}$ more stable than the perpendicular conformation of $T_{1}$ at the 90 double bond rotation ($T_{1}^{\text{perp}}$, see Figure 3). In fact, this represents the energetic hurdle for isomerization. Once the perpendicular geometry is, however, accessed, rapid relaxation to $S_{0}$ via reverse ISC becomes likely due to the significantly higher spin‐orbit coupling (30 ${\mathrm{cm}}^{-1}$ versus ∼0 ${\mathrm{cm}}^{-1}$ in the planar geometry). This shifts the equilibrium toward the perpendicular triplet structure, enabling the *E*‐to‐*Z* photoisomerization.

Overall, our findings show directly that photoisomerization of **1b**‐*E* occurs exclusively from the triplet excited state. The dynamics of the stepwise process can be quantified with high precision, owing to the pronounced spectral separation of species in the transient data. Further, the elucidated dynamics align with literature reports on the *E*‐to‐*Z* isomerization of structurally related thioindigo systems, where a long‐lived *E*‐triplet state has been observed.^[^ ^53^ ^]^ It is widely accepted that isomerization in such systems proceeds exclusively via the $T_{1}$ pathway: the rate of product formation matches that of the $T_{1}$ decay,^[^ ^36^, ^53^ ^]^ and oxygen quenching thereof effectively suppresses photoisomerization.^[^ ^32^ ^]^ In the case of **1b**, however, no pronounced influence of oxygen in the air was noticeable,^[^ ^25^ ^]^ which made a triplet mechanism not extremely likely from the outset. Although fs‐TA experiments revealed a short‐lived absorption, which was assigned as a $S_{1}\to S_{n}$ ESA, it could be shown that the singlet is not the productive pathway to **1b**‐*Z*.

#### *Z*‐to‐*E* Photoisomerization

To investigate the *Z*‐to‐*E* photoisomerization pathway, the **1b**‐*Z* isomer was generated by continuous LED irradiation at 750 nm. Subsequently, TA measurements were performed to track the excited‐state dynamics following photoexcitation of **1b**‐*Z* (see Figure 5a).

Upon 550 nm excitation, the femtosecond TA spectra of **1b**‐*Z* in tetrahydrofuran reveal an immediate GSB ranging from 550 to 750 nm, reflecting the position and spectral shape of the ground state absorption of **1b**‐*Z* (see filled curve in Figure 5a).

This GSB is accompanied by several ESA features. These include a prominent maximum centered at 925 nm, a weak shoulder near 1000 nm, and broad, weak signals extending from 1100 to 1300 nm. After 8 ps, new ESAs emerge between 600 and 750 nm, partially overlapping with the GSB, and the ESA at 925 nm becomes spectrally narrower. This intermediate decays within 17 ps, resulting in a recovery of the GSB between 550 and 750 nm. Over the next 154 ps, the 925 nm ESA undergoes a red‐shift to 930 nm, while the broad ESA features beyond 1100 nm vanish. The 930 nm ESA persists with a lifetime of approximately 31 ns, as measured by means of nanosecond TA spectroscopy. Its decay is followed by the appearance of new features in the positive signal region between 650 and 750 nm. These final spectral features coincide with the steady‐state absorption of the *E*‐isomer and are long‐lived (infinite), indicating successful formation of **1b**‐*E* as the photoproduct. For comparison, a differential spectrum between the ground state absorption spectra of the *E*‐ and *Z*‐isomers of **1b** is shown as a green, filled curve in Figure 5b.

Global target analysis revealed four characteristic time constants: 8 ps, 17 ps, 154 ps, and 31 ns next to the infinite component (see Figure 5b). The spectral evolution associated with the 8 ps component is characterized by a decay of ESAs in the 960 to 1100 nm range, accompanied by a sharpening and blue shift of the primary ESA. This transformation results in the emergence of a sharp, unstructured band centered at 920 nm. We attribute this process to a vibrational cooling and a flattening of the initially distorted, step‐like geometry of **1b**‐*Z* in $S_{1}$. This interpretation is supported by ADC(2) simulations, which predict a blue shift in $S_{1}\to S_{n}$ absorptions upon planarization of the Franck–Condon geometry (see Figure 5c).

Further insights from theory reveal the presence of two distinct local minimum geometries on the $S_{1}$ potential energy surface. They both exhibit planar or near‐planar geometries (see gray vs. light‐gray geometry in Figure 3c). Nonetheless, these minima differ markedly in their electronic character, as evidenced by their simulated transient absorption spectra (cf. Figure 5c). The fully flattened structure, denoted $S_{1}^{\min,1}$ (light‐gray geometry in Figure 3c), exhibits a dominant ESA at 756 nm ($S_{1}\to S_{4}$, f=0.115). In contrast, the second structure, $S_{1}^{\min,2}$ (a flattened but twisted conformation; gray geometry in Figure 3c), displays an intense ESA at 893 nm ($S_{1}\to S_{5}$, f=0.418). Both excitations correspond to charge‐transfer transitions, in which the electron density shifts from the central thioindigo toward the anthracene. For $S_{1}^{\min,1}$, the accepting orbital is localized in proximity to the oxygen atoms of the dye, while in $S_{1}^{\min,2}$, it is fully delocalized across the anthracenes (see charge density differences in Figure 5e,f, respectively).

Given that the global target analysis required a branching pathway to adequately describe the data, the 8 ps time constant is assigned to the population of two distinct local minima on the $S_{1}$ surface: the conformers configured with planar $S_{1}^{\min,1}$ and the twisted $S_{1}^{\min,2}$ *Z*‐configured conformers.

Subsequently, one of these $S_{1}s$ undergoes non‐radiative decay back to $S_{0}$ with a characteristic time of 16 ps. This process is evident in the decay of the ESA features ranging from 600 to 750 nm and partial GSB recovery between 500 and 600 nm (see 16 ps component in Figure 5b,c). The respective evolution‐associated spectrum coincides with the simulated TA spectrum of $S_{1}^{\min,1}$ and thus is tentatively assigned to the non‐radiative relaxation of the planarized geometry. Such ultrafast $S_{1}\to S_{0}$ relaxation has also been observed in related (hemi‐)thioindigo systems, where efficient internal conversion via accessible conical intersections enables decay on the picosecond timescale.^[^ ^42^, ^43^, ^48^ ^]^

In parallel, the twisted conformer $S_{1}^{\min,2}$ undergoes ISC to $T_{1}$ within 154 ps. This ISC event is accompanied by a red shift of the 925 nm ESA and a concurrent loss of ESA signals in the 1100–1300 nm region, mirroring the behavior observed for **1b**‐*E*.

Finally, $T_{1}$ undergoes photoisomerization to the *E*‐isomer on a timescale of 31 ns. This transformation is marked by the emergence of new ESAs between 650 and 750 nm, consistent with the ground‐state absorption of the *E*‐isomer (Figure 5b).

The mechanism of *Z*‐to‐*E* photoisomerization in thioindigoids has long been debated. Although early studies agree on the involvement of a triplet intermediate,^[^ ^34^, ^35^, ^36^, ^37^ ^]^ they diverge on whether this species shares the same geometry as the *E*‐to‐*Z* intermediate or represents a distinct, *Z*‐configured triplet. Some even proposed that both singlet and triplet pathways may contribute. Our combined experimental and computational analysis for the PAT photoswitch now provides a clear picture: **1b**‐*Z* undergoes $T_{1}$‐mediated isomerization similar to the *E*‐isomer, but with an additional ultrafast deactivation channel. Our insights reveal branching on the surface of $S_{1}$ into a planar minimum ($S_{1}^{\min,1}$) that relaxes to the ground state and a twisted conformer ($S_{1}^{\min,2}$) that undergoes ISC to $T_{1}$ before completing the isomerization. This dual‐pathway mechanism reconciles earlier hypotheses, confirming that while the triplet state governs very efficient photoisomerization, the singlet state plays a crucial competing role in deactivation.

## Conclusion

We have presented a comprehensive investigation of the photoisomerization mechanisms of the NIR‐responsive *peri*‐anthracenethioindigo (PAT) photoswitch, combining excited‐state quantum chemical calculations with femtosecond and nanosecond transient absorption spectroscopy. Owing to its highly favorable spectroscopic properties, PAT enables temporally and spectrally well‐resolved tracking of the underlying excited‐state processes. This allows for a quantitative mechanistic picture of the pathways that govern its efficient and selective photoisomerization behavior.

Our results demonstrate that *E*‐to‐*Z* isomerization from the singlet excited state is energetically disfavored, and that productive switching proceeds instead via the triplet manifold. The same triplet intermediate also mediates the reverse *Z*‐to‐*E* isomerization. In addition, we identify a competing, unproductive singlet‐state pathway in the latter direction, offering a strategic handle for rational design—for example, through incorporation of heavy‐atom substituents (e.g., Cl, Br, I) to enhance intersystem crossing to the productive triplet state. Importantly, the $T_{1}$‐$S_{0}$ gap of PAT (for $T_{1}^{E}$: 0.72 eV) lies below the energy of molecular oxygen (∼1 eV), thus minimizing quenching and enabling efficient photoswitching under ambient conditions without the need for oxygen exclusion. Additionally, the relatively short life‐time of the PAT triplet states is likely to further minimize the efficiency of oxygen sensitization. The unique structural features of PAT, particularly the *peri*‐substitution pattern and conjugation extension via ring annulation, emerge as key determinants of its well‐defined and favorable photochemical performance.

This study therefore provides crucial fundamental insights into the excited‐state dynamics of a distinct class of thioindigo‐based switches and establishes a blueprint for the future design of high‐performance, NIR‐active photoswitches with predictable and tunable properties.

## Conflict of Interests

The authors declare no conflict of interest.

## Supporting information

Supporting Information

## Data Availability

The data that support the findings of this study are openly available in CompPhotoChem/PAT_mechanism at https://doi.org/10.5281/zenodo.15413053 , reference number 15413054.
