# Supplementary material for: From Triplet to Twist: The Photochemical E/Z‐Isomerization Pathway of the Near‐Infrared Photoswitch peri‐Anthracenethioindigo
Source: Angew Chem Int Ed Engl. 2025 Aug 7;64(38):e202510626. doi: 10.1002/anie.202510626 (PMC12435429; doi:10.1002/anie.202510626)
Supplement: Supplementary file 1 — Supporting Information [file ANIE-64-e202510626-s001.pdf]

Supporting Information

From Triplet to Twist: The Photochemical *E/Z*-Isomerization Pathway of the Near-Infrared Photoswitch *peri*-Anthracenethioindigo

Martina Hartinger[1,†], Maximilian Herm[2,†], Christoph Schüßlbauer[2], Laura Köttner[3], Dirk Guldi[2,*], Henry Dube[3,*], Carolin Müller[1,*]

[1]Friedrich-Alexander-Universität Erlangen-Nürnberg, Computer Chemistry Center, Nägelsbachstraße 25, 91052 Erlangen

[2]Friedrich-Alexander-Universität Erlangen-Nürnberg, Egerlandstraße 3, 91054 Erlangen

[3]Friedrich-Alexander-Universität Erlangen-Nürnberg, Nikolaus-Fiebiger Straße 10, 91054 Erlangen

[†]These authors contributed equally.

[*]dirk.guldi@fau.de, henry.dube@fau.de, carolin.cpc.mueller@fau.de

Contents

1 Methods **2**
1.1 Computational Details 2
1.2 Experimental Details 3

2 Stationary Points **3**
2.1 Ground State Minima 3
2.2 Excited-State Minima 7
2.3 Spin-Orbit Couplings 11

3 Experimental and simulated Transient Absorption Data **12**
3.1 E-to-Z-isomerization 12
3.2 Z-to-E-isomerization 13

4 References **14**

1 Methods

All optimized geometries and simulated spectroscopic properties as well as the experimental transient absorption spectra can be found on Zenodo [1].

1.1 Computational Details

For our computations, we chose a static theoretical approach, allowing us to combine structural information from static theory with dynamic information from experiments. This approach has the advantage that experiment and theory not only support but complement each other. We are aware of the limitations of using static calculations to draw conclusions about the dynamics of our systems, however, due to the size of PAT with 90 atoms, state-of-the-art non-adiabatic molecular dynamics (NAMD) simulations become prohibitively expensive. Besides the size of the molecule, a key issue is potential involvement of long-lived triplet states in the isomerization of thioindigoid switches, which require simulation times up to nano- or even microseconds instead of a few picoseconds as in routine-NAMD studies [2–6].

Our study combines density functional theory (DFT), time-dependent DFT (TD-DFT) and algebraic diagrammatic construction theory of second order (ADC(2)) calculations to investigate the ground- and excited-state properties of the full PAT molecule (**1b**) and its simplified analogue (**1a**), in which mesityl groups are replaced by hydrogen atoms to reduce computational cost. To minimize computational costs in the ADC(2) calculations, these were only performed for **1a**, since our TD-DFT excitation analysis on **1b** revealed no participation of the residues in the excitation process. All (TD-)DFT calculations, however, were conducted on the **1b**. Solvent effects were modeled using the solute electron density variant of the integral equation formalism of the polarizable continuum model (IEFPCM) [7, 8] of benzene for DFT-based methods and the conductor-like screening model (COSMO) [9] of tetrahydrofuran for ADC(2) calculations. ADC(2) calculations were performed using Turbomole 7.7 [10], DFT and TD-DFT calculations were performed in Gaussian 16 [11] and spin-orbit couplings (SOCs) in ORCA [12, 13].

Stationary Geometries. Geometries of **1b** were optimized for the ground state (S_0), first singlet excited state (S_1), and first triplet state (T_1) by means of density functional theory (DFT) and time-dependent DFT (TD-DFT) using the B3LYP [14] functional and employing the triple-ζ 6-311G(d,p) [15–17] basis set for all atoms. The identified stationary points include the ground state geometries of **1b**-*E* (S_0^E) and **1b**-*Z* (S_0^Z), as well as several local minima on the S_1 (S_1^E, $S_1^{Z,1}$, $S_1^{Z,2}$) and T_1 (T_1^E, T_1^{perp}) potential energy surfaces. The respective geometries of **1a** were obtained by replacing the mesityl-groups of the local minimum geometries of **1b** with hydrogen atoms and re-optimizing the structures at (TD-)DFT level of theory.

Relaxed Potential Energy Surface Scans. To asses potential photosimoerization in the T_1 state, we performed a relaxed scan in the T_1 state along the torsion angle of the central double bond using the above described DFT protocol, B3LYP/6-311G(d,p). While, structures were relaxed on the T_1 potential energy surface, all other energies at these points were obtained *via* single-point calculations. Spin-orbit couplings (SOCs) along the scan and at other stationary points were calculated with the same B3LYP protocol.

Transient Absorption Spectra. Vertical excitation energies and oscillator strengths for ground-state singlet-singlet ($S_0 \rightarrow S_n$) were calculated using both TD-DFT (B3LYP/6-311G(d,p)) and and the algebraic diagrammatic construction theory of second order (ADC(2)/def2-TZVP [18]). For TD-DFT, the lowest 90 excited states were included, while ADC(2) calculations considered the lowest 10 states of the respective multiplicity.

Excited-state absorption from S_1 or T_1 to higher singlet or triplet states, *i.e.*, $S_1 \rightarrow S_n$) or $T_1 \rightarrow T_n$ transitions, were computed for at the same ADC(2) level, also including the lowest 10 singlet excited states. To this end, stationary points on the S_1 and T_1 surface as well as the Franck-Condon geometries of the *E*- and *Z*-isomers of **1a** and **1b** were take into account. For **1a** these were computed using ADC(2)/def2-TZVP [18] level of theory, which has very recently been shown to provide excited-state absorption energies and oscillator strengths in good agreement with reference values calculated at the QR-CC3 level of theory [19]. Additionally,

$T_1 \rightarrow T_n$ transitions were calculated for **1a** and **1b** at the TD-B3LYP/6-311G(d,p)/IEFPCM(THF) level of theory, where the lowest 90 excited states were included.

With the calculated ground- and excited-state absorption data, *i.e.* vertical excitation energies and oscillator strengths, we simulated transient absorption spectra for various geometries assuming population of different potentially involved states. To this end, we subtracted the absorption of the ground-state equilibrium geometries ($S_0 \rightarrow S_n$) from excited state absorption ($S_1 \rightarrow S_n$ or $T_1 \rightarrow T_n$) of a specific state.

1.2 Experimental Details

Synthesis. Compound **1b** was re-synthesized following the instructions as previously reported by some of us [20].

Transient Absorption (TA) Spectroscopy. Ultrafast pump-probe transient absorption (fsTA) spectroscopy was performed using an Astrella-F-1K amplified Ti:sapphire femtosecond laser system from Coherent, operating at a repetition rate 1 kHz, 5.5 W power (5 mJ pulse energy), pulse duration of 80 fs. To acquire the fsTA spectra an Ultrafast Systems HELIOS TA spectrometer was used with time delays from 0 to 7200 ps. White light for the probing pulse in the visible region of the optical spectrum (≈420–770 nm) was generated by focusing part of the fundamental 800 nm output onto a 2 mm sapphire disk. For (near) IR (800–1350 nm) white light, a 10 mm sapphire was used. For nanosecond TA (nsTA) experiments, a photonic crystal fiber supercontinuum laser with a 1064 nm fundamental was used with an EOS spectrometer to record TA spectra between time delays ranging from 1 ns to 400 μs.

For both fs- and ns-experiments, the excitation wavelengths (750 nm and 550 nm) were generated *via* a TOPAS Prime from Light Conversion with standard NirUVis extension. For the measurements the energy per pump pulse was tuned to 500 nJ. All samples were prepared at room temperature in 2×10 mm quartz cuvettes, purged with argon for 20 min and stirred during the measurements. For the fsTA measurements of the *Z*-to-*E* isomerization a flow cell was used employing a KDS100 Legacy syringe pump from Fisher Scientific.

Target analysis of the fsTA data was performed using GloTarAn 1.5.1 [21], which is a free, Java-based graphical user interface to the R-package TIMP. Pre-processing steps included the modelling of the instrument response function (IRF) and dispersion (chirp of the white light pulse) correction, moreover the nsTA data were corrected for scattered light.

2 Stationary Points

2.1 Ground State Minima

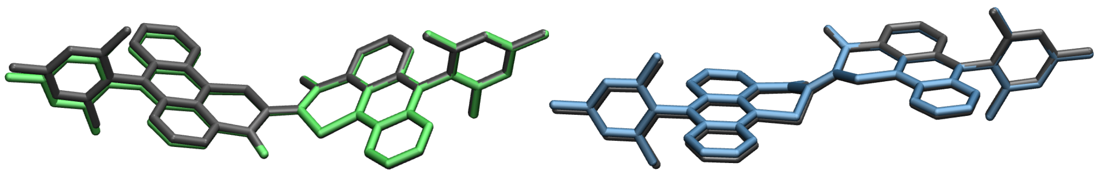

(a) 0.28 Å **(b)** 0.22 Å

Figure S1: Comparison of the equilibrium geometries of S_0^E (a) and S_0^Z (b) as obtained at B3LYP/6-311G(d,p)/IEFPCM(C_6H_6) level of theory including Grimme's D3 dispersion correction with Becke-Johnson damping [22] (gray) and without dispersion correction (colored geometries). The RMSD values between the two structures are indicated by the numbers in the subcaption.

Table S1: Vertical transition energies (ΔE) and oscillator strengths (f) for the $S_0 \rightarrow S_1$ excitation for the *E*- and *Z*-isomers of **1a** and **1b**, obtained by means of TD-DFT and ADC(2) calculations. Solvent effects were taken into account by means of implicit solvent models for benzene. The two rows labelled with "D3BJ optimized geometries" use the isomers optimized including D3BJ correction (see Figure S1), whereas all other rows use isomers optimized without D3BJ, as used throughout the manuscript.

compound		method	ΔE	f	f(E)/f(Z)	character
E-isomer	**1a**	B3LYP/6-311G(d,p)	1.66	0.63	1.4	H $\rightarrow$ L, $\pi\pi^*$
	**1a**	ADC(2)/def2-TZVP	1.85	0.45	1.3	H $\rightarrow$ L, $\pi\pi^*$
	**1b**	CAM-B3LYP/6-311G(d,p)	2.03	0.97	1.1	H $\rightarrow$ L, $\pi\pi^*$
	**1b**	B3LYP/6-311G(d,p)	1.61	0.78	1.5	H $\rightarrow$ L, $\pi\pi^*$
	**1b**	B3LYP/6-311G(d,p), D3BJ optimized geometries	1.61	0.76	1.4	H $\rightarrow$ L, $\pi\pi^*$
	**1b**	Experiment	1.77	–	1.1	–
Z-isomer	**1a**	B3LYP/6-311G(d,p)	1.98	0.46	1.4	H $\rightarrow$ L, $\pi\pi^*$
	**1a**	ADC(2)/def2-TZVP	2.35	0.36	1.3	H $\rightarrow$ L, $\pi\pi^*$
	**1b**	CAM-B3LYP/6-311G(d,p)	2.50	0.85	1.1	H $\rightarrow$ L, $\pi\pi^*$
	**1b**	B3LYP/6-311G(d,p)	1.95	0.57	1.5	H $\rightarrow$ L, $\pi\pi^*$
	**1b**	B3LYP/6-311G(d,p), D3BJ optimized geometries	1.97	0.53	1.4	H $\rightarrow$ L, $\pi\pi^*$
	**1b**	Experiment	2.10	–	1.1	–

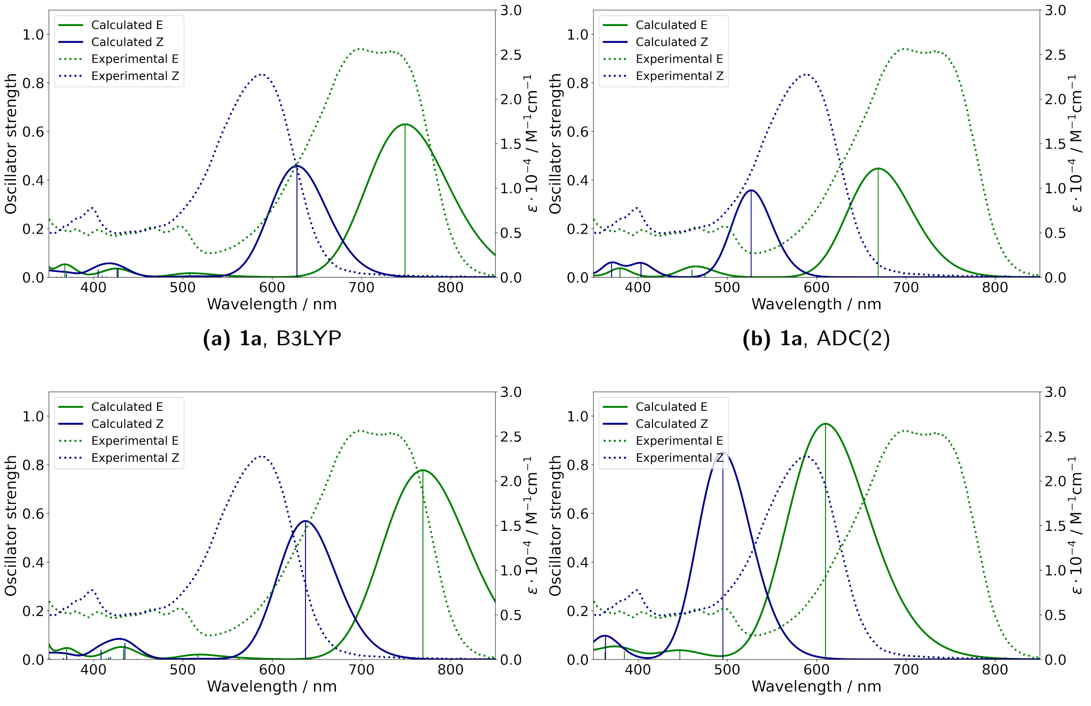

(a) **1a**, B3LYP

(b) **1a**, ADC(2)

(c) **1b**, B3LYP

(d) **1b**, CAM-B3LYP

Figure S2: Comparison of the experimental absorption spectra **1b**-*E* (green, dashed) and **1b**-*Z* (blue, dashed) to the simulated absorption spectra of **1a** (top row) and **1b** (bottom row). Simulations were performed by means of ADC(2)/def2-TZVP/COSMO(THF) (a) and TD-DFT/6-311G(d,p)/IEFPCM(benzene) employing the B3LYP (a, c) or CAM-B3LYP (b) functional.

Table S2: Simulated vertical excitation energies, oscillator strengths (f) and charge density differences (CDDs) of some singlet states involved in the photoexcitation of **1a**- and **1b**-*E*. The table comprises selected singlet-singlet transitions for the ground state equilibrium geometry (S_0^{FC}). Excitation occurs from white to black ($\rho = \pm 0.0015$).

**1b**-*E*, TD-DFT/6-311G(d,p)/IEFPCM(C_6H_6)		
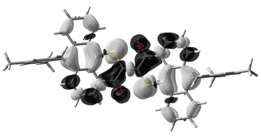	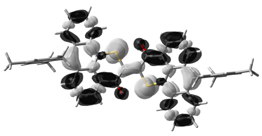	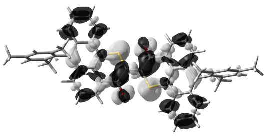
S_1<br>1.61 eV (641 nm)<br>$f = 0.78$	S_3<br>2.40 eV (516 nm)<br>$f = 0.02$	S_5<br>2.86 eV (434 nm)<br>$f = 0.04$
**1a**-*E*, TD-DFT/6-311G(d,p)/IEFPCM(C_6H_6)		
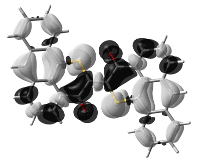	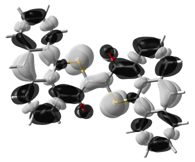	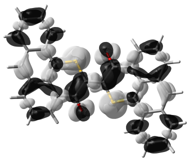
S_1<br>1.66 eV (749 nm)<br>$f = 0.63$	S_3<br>2.44 eV (508 nm)<br>$f = 0.02$	S_5<br>2.90 eV (428 nm)<br>$f = 0.03$
**1a**-*E*, ADC(2)/def2-TZVP/COSMO(THF)		
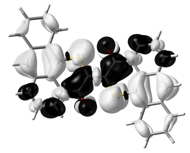	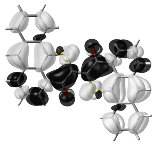	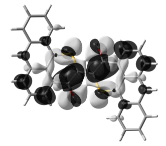
S_1<br>1.77 eV (700 nm)<br>$f = 0.48$	S_2<br>2.49 eV (498 nm)<br>$f = 0.02$	S_3<br>2.75 eV (451 nm)<br>$f = 0.03$

Table S3: Simulated vertical excitation energies, oscillator strengths (f) and charge density differences (CDDs) of some singlet states involved in the photoexcitation of **1a**- and **1b**-*Z*. The table comprises selected singlet-singlet transitions for the ground state equilibrium geometry (S_0^{FC}). Excitation occurs from white to black ($\rho = \pm 0.0015$).

**1b**-*Z*, TD-DFT/6-311G(d,p)/IEFPCM(C_6H_6)		
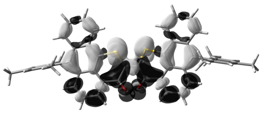	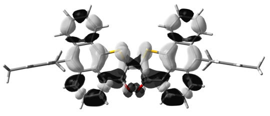	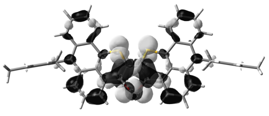
S_1	S_4	S_6
1.95 eV (637 nm)	2.85 eV (435 nm)	3.04 eV (409 nm)
$f = 0.57$	$f = 0.07$	$f = 0.04$
**1a**-*Z*, TD-DFT/6-311G(d,p)/IEFPCM(C_6H_6)		
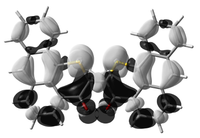	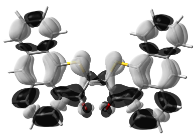	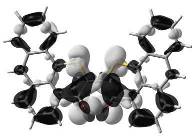
S_1	S_4	S_6
1.98 eV (628 nm)	2.91 eV (427 nm)	3.06 eV (405 nm)
$f = 0.46$	$f = 0.04$	$f = 0.03$
**1a**-*Z*, ADC(2)/def2-TZVP/COSMO(THF)		
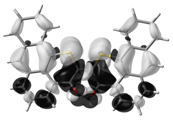	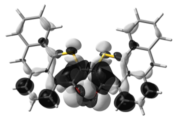	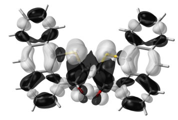
S_1	S_4	S_5
2.23 eV (556 nm)	3.15 eV (394 nm)	3.29 eV (377 nm)
$f = 0.39$	$f = 0.06$	$f = 0.09$

2.2 Excited-State Minima

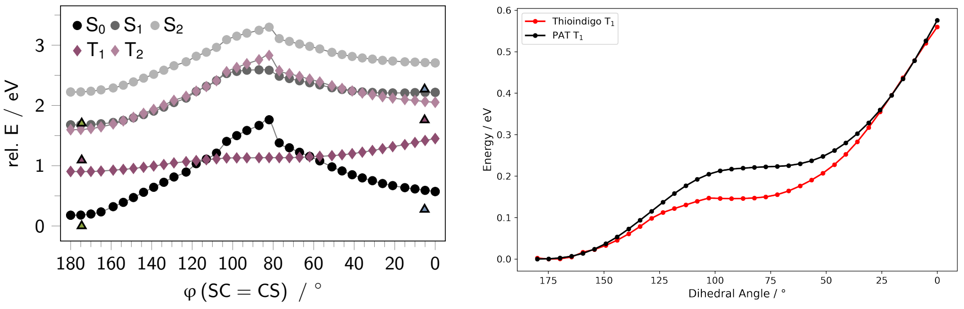

(a) **(b)**

Figure S3: a) Relaxed scan of **1b** on the T_1 potential energy surface and respective energies of the three lowest singlet states (S_0, S_1, S_2, circle symbols) and a higher lying triplet state (T_2, light-purple). The energies of the Franck–Condon point energies (i.e. S_0^E and S_0^Z) are indicated by triangles. b) Relaxed scan on the T_1 surface for **1b** (black) and thioindigo (red). All energies and geometries were obtained at (TD-)B3LYP/6-311G(d,p)/IEFPCM(benzene) level of theory. Energies are given relative to the respective energetic minimum along the scan coordinate.

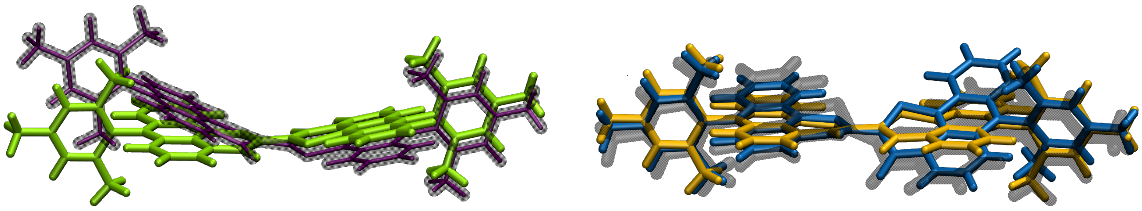

(a) S_0^E (0.00 eV, black), S_1^E (1.41 eV, purple) and T_1^E (0.78 eV, green)

(b) S_0^Z (0.23 eV, black), $S_1^{min,1}$ (2.29 eV, orange) and $S_1^{min,2}$ (1.87 eV, blue)

Figure S4: Comparison of the local minimum geometries S_0^E (a, black), S_1^E (a, purple) and T_1^E (a, green) as well as S_0^Z (b, black), $S_1^{min,1}$ (b, orange) and $S_1^{min,2}$ (b, blue). The minima were optimized for structure **1b** at TD-B3LYP/6-311G(d,p)/IEFPCM(C_6H_6) level of theory. The relative energies of the respective geometries are given in the parenthesis (in eV, relative to S_0^E).

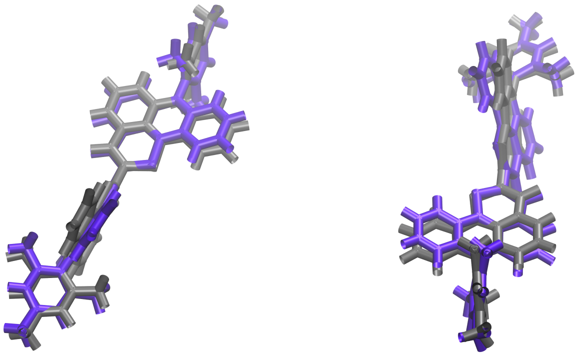

Figure S5: Minimum energy crossing point between T_1 and S_0 obtained at B3LYP/6-311G(d,p)/CPCM(benzene) level of theory (purple). For comparison, the geometry of the minimum geometry T_1^{perp} is shown in gray.

Table S4: Simulated vertical excitation energies, oscillator strengths (f) and charge density differences (CDDs) of selected $S_1 \rightarrow S_n$ excited state absorption features of **1a**. The table comprises selected singlet-singlet excited state transitions for the Franck-Condon geometry of the *E*- and *Z*-isomer (S_0^E and S_0^Z). Excitation occurs from white to black ($\rho = \pm 0.0015$). The excitations are computed at ADC(2)/def2-TZVP/COSMO(THF) level of theory.

$S_1 \rightarrow S_n$ for **1a**-*E* (Franck-Condon geometry)	
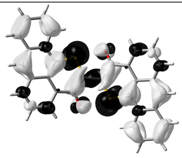	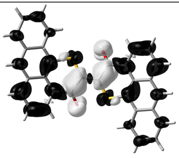
S_2	S_4
0.72 eV (1722 nm)	1.10 eV (1127 nm)
$f = 0.22$	$f = 72$
$S_1 \rightarrow S_n$ for **1a**-*Z* (Franck-Condon geometry)	
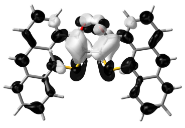	
S_3	
0.90 eV (1378 nm)	
$f = 0.59$	

Table S5: Simulated vertical excitation energies, oscillator strengths (f) and charge density differences (CDDs) of selected $S_1 \to S_n$ excited state absorption features of **1a**-*Z*. The table comprises selected singlet-singlet excited state transitions for the Z-configured minima on S_1, namely $S_1^{min,1}$ and $S_1^{min,2}$. Excitation occurs from white to black ($\rho = \pm 0.0015$). The excitations are computed at ADC(2)/def2-TZVP/COSMO(THF) level of theory.

$S_1 \to S_n$ for **1a**-*Z* ($S_1^{min,1}$)	
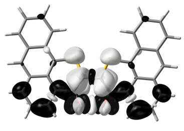	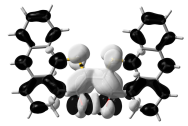
S_4	S_8
1.64 eV (756 nm)	2.39 eV (518 nm)
$f = 0.12$	$f = 0.33$
$S_1 \to S_n$ for **1a**-*Z* ($S_1^{min,2}$)	
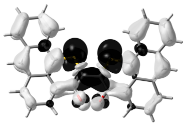	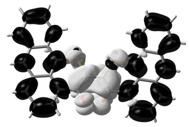
S_2	S_5
0.82 eV (1512 nm)	1.38 eV (898 nm)
$f = 0.41$	$f = 0.42$

Table S6: Simulated vertical excitation energies, oscillator strengths (f) and charge density differences (CDDs) of selected $T_1 \to T_n$ excited state absorption features of **1a**. The table comprises selected triplet-triplet transitions for two local minima on T_1, namely T_1^E and T_1^{perp}. Excitation occurs from white to black ($\rho = \pm 0.0015$). The excitations are computed at ADC(2)/def2-TZVP/COSMO(THF) level of theory.

$T_1 \to T_n$ for **1a**-*E* (T_1^E)	
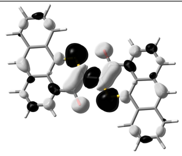	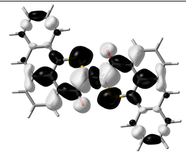
T_5	T_8
1.52 eV (816 nm)	1.99 eV (623 nm)
$f = 1.03$	$f = 0.13$
$T_1 \to T_n$ for **1a** (T_1^{perp})	
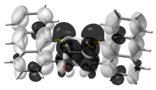	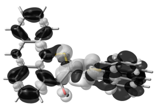
T_3	T_6
1.99 eV (623 nm)	2.59 eV (479 nm)
$f = 0.13$	$f = 0.39$

Table S7: Simulated vertical excitation energies, oscillator strengths (f) and charge density differences (CDDs) of selected $T_1 \rightarrow T_n$ excited state absorption features of **1a**. The table comprises selected triplet-triplet transitions for two local minima on T_1, namely T_1^E and T_1^{perp}. Excitation occurs from white to black ($\rho = \pm 0.0015$). The excitations are computed at TD-B3LYP/6-311G(d,p)/IEFPCM(C_6H_6) level of theory.

$T_1 \rightarrow T_n$ for **1a**-*E* (T_1^E)	
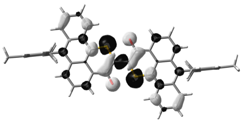	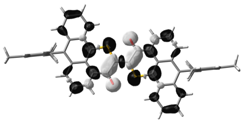
T_2<br>0.76 eV (1631 nm)<br>$f = 0.10$	T_4<br>1.33 eV (932 nm)<br>$f = 0.96$
$T_1 \rightarrow T_n$ for **1a** (T_1^{perp})	
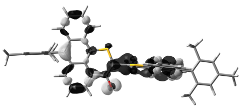	
T_6<br>1.98 eV (626 nm)<br>$f = 0.58$	

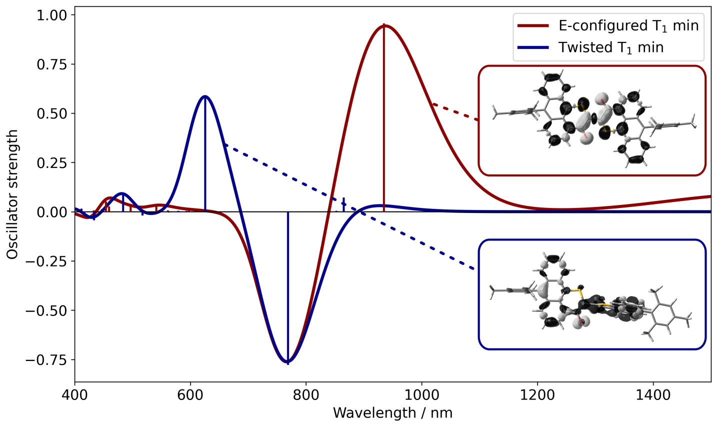

Figure S6: Simulated transient absorption spectra for a $S_0^E \rightarrow T_1$ excitation as obtained by means of TD-B3LYP/6-311G(d,p)/IEFPCM(C_6H_6).

2.3 Spin-Orbit Couplings

Table S8: Spin-orbit couplings (SOCs) between state 1 (T_1 or T_2) and state 2 (S_0 or S_1) of selected stationary points, *i.e.*, local minimum geometries in the ground- (S_0) and excited states (S_1 and T_1). The SOCs are computed at TD-B3LYP/6-311G(d,p)/PCM(benzene) and are reported in cm^{-1}. The values in the parenthesis indicate the energy differences (in eV) between state 1 and state 2 (positive values indicate $E(T_n) > E(S_n)$).

		E-configuration				*Z*-configuration		
1	2	S_0^E	S_1^E	T_1^E	T_1^{perp}	S_0^Z	$S_1^{min,1}$	$S_1^{min,2}$
T_1	S_0	7.62 (+0.95)	6.87 (+0.66)	0.01 (+0.54)	30.47 (−0.45)	15.04 (+1.48)	12.73 (+0.64)	31.23 (+0.34)
T_1	S_1	0.06 (−0.65)	0.01 (−0.73)	0.01 (−0.81)	1.71 (−1.45)	0.69 (−0.51)	2.62 (−0.35)	3.02 (−0.98)
T_2	S_1	2.14 (−0.17)	0.99 (−0.10)	0.00 (−0.08)	0.68 (+0.12)	0.77 (−0.30)	11.96 (−0.01)	4.89 (−0.05)

3 Experimental and simulated Transient Absorption Data

3.1 E-to-Z-isomerization

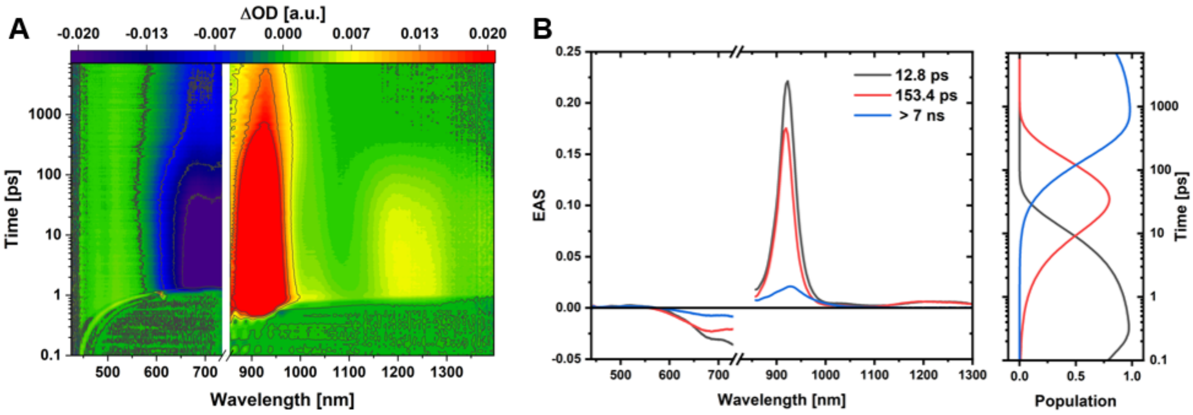

Figure S7: (A) Fs-differential absorption spectra of E-PAT at room temperature in argon purged THF at time delays between 1 and 7200 ps after 750 nm laser photoexcitation. (B) Evolution-associated spectra (EAS) obtained via global analysis, applying a sequential deactivation model, and corresponding population graph.

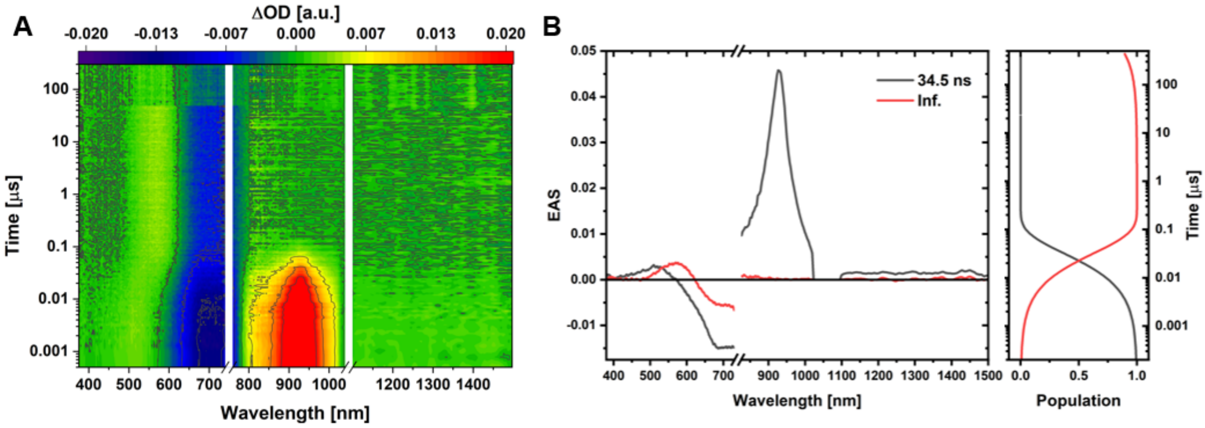

Figure S8: (A) Ns-differential absorption spectra of E-PAT at room temperature in argon purged THF at time delays between 1 and 7200 ps after 750 nm laser photoexcitation. (B) Evolution-associated spectra (EAS) obtained via global analysis, applying a sequential deactivation model, and corresponding population graph.

3.2 Z-to-E-isomerization

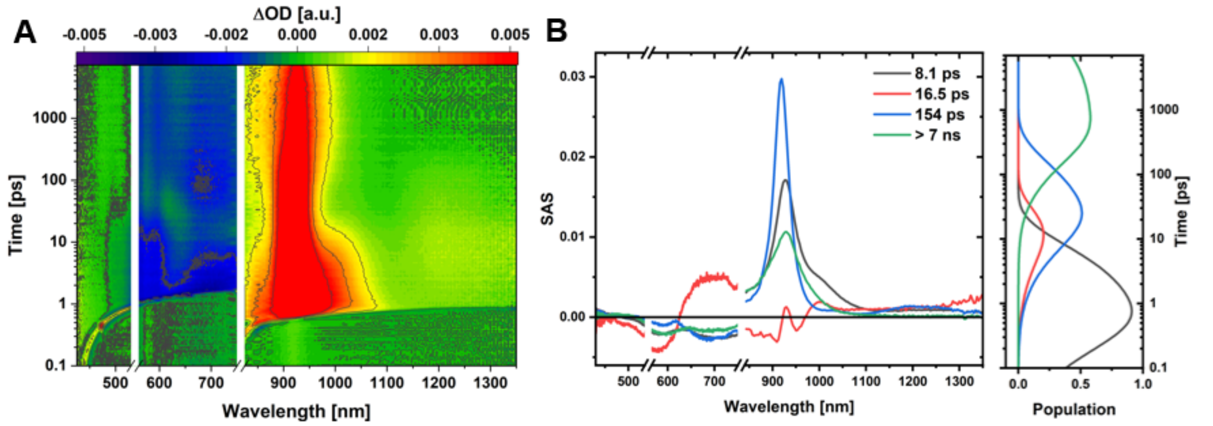

Figure S9: (A) Fs-differential absorption spectra of Z-PAT at room temperature in argon purged THF at time delays between 1 and 7200 ps after 550 nm photoexcitation. (B) Species-associated spectra (SAS) obtained via target analysis, applying a parallel deactivation model, and corresponding population graph.

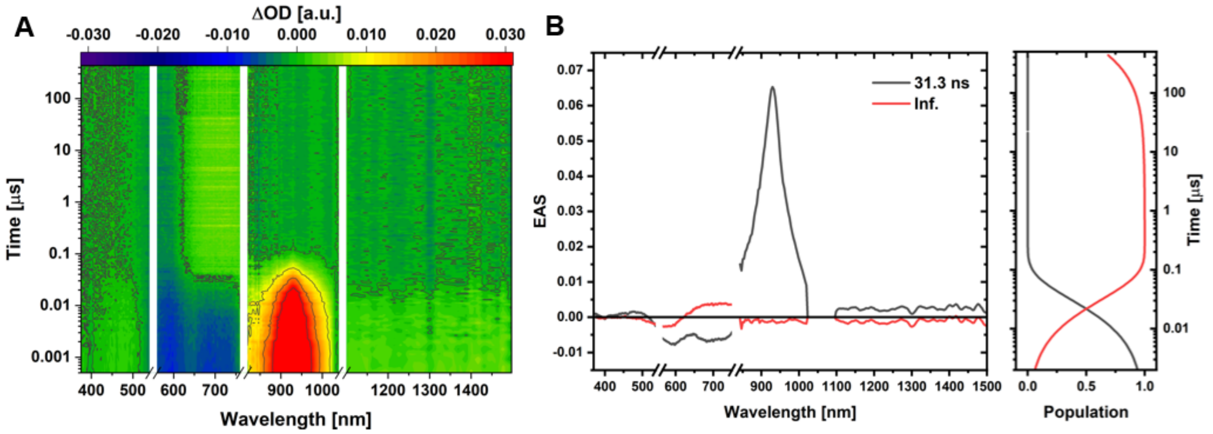

Figure S10: (A) Ns-differential absorption spectra of Z-PAT at room temperature in argon purged THF at time delays between 1 and 7200 ps after 550 nm photoexcitation. (B) Evolution-associated spectra (EAS) obtained via global analysis, applying a sequential deactivation model, and corresponding population graph.

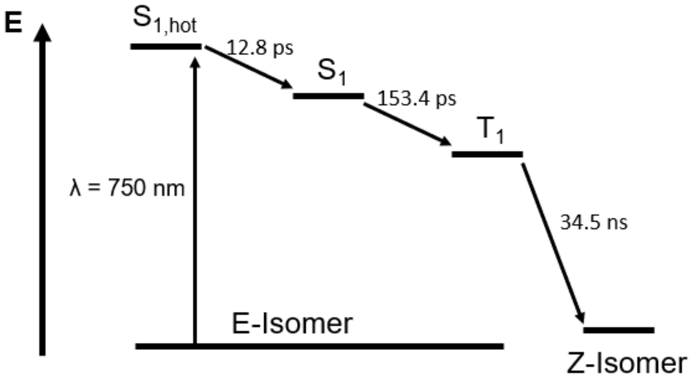

Figure S11: Kinetic scheme of the E-Z photoisomerization upon 750 nm photoexcitation in THF.

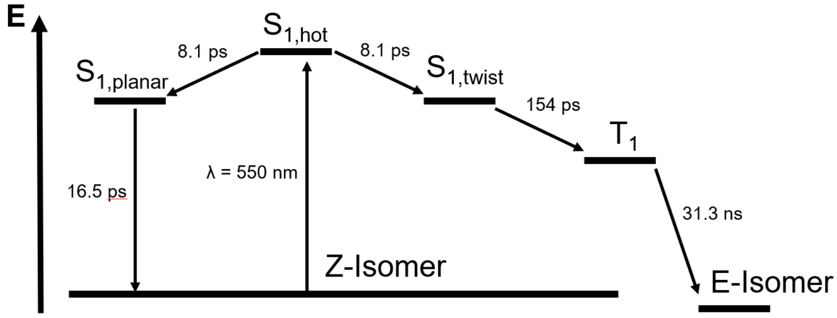

Figure S12: Kinetic scheme of the *Z*-to-*E* photoisomerization of **1b** at 550 nm photoexcitation in THF.

4 References

[1] M. Hartinger, M. Herm, C. Schüßlbauer, L. Köttner, D. Guldi, H. Dube, C. Müller: "CompPhotoChem / PAT_mechanism: experiment + theory", *Zenodo* **2025**, DOI 10.5281/zenodo.15480125.

[2] K. Grellmann, P. Hentzschel: "Mechanism of the photochemical cis⇄trans isomerization of thioindigo and 6, 6'-diethoxy-thioindigo in solution", *Chemical Physics Letters* **1978**, *53*, 3, 545–551, DOI 10.1016/0009-2614(78)80066-9.

[3] S. Krysanov, M. Alfimov: "cis⇄trans photoisomerization of thioindigoid dyes studied by picosecond flash photolysis", *Chemical Physics Letters* **1981**, *82*, 1, 51–54, DOI 10.1016/0009-2614(81)85104-4.

[4] S. Krysanov, M. Alfimov: "Picosecond Laser Study of Thioindigoid Dyes Photoisomerization", *Laser Chemistry* **1984**, *4*, 1-6, 121–128, DOI 10.1155/LC.4.121.

[5] T. Karstens, K. Kobs, R. Memming, F. Schroppel: "Photoisomerization mechanism of thioindigo dyes. Thioindigo in nonpolar solvents", *Chem. Phys. Letters* **1977**, *48*, 3, 540–544, DOI 10.1016/0009-2614(77)85088-4.

[6] R. Memming, K. Kobs: "Cis→trans-photoisomerization of thioindigo", *Berichte der Bunsengesellschaft für physikalische Chemie* **1981**, *85*, 3, 238–242, DOI 10.1002/bbpc.19810850313.

[7] S. Miertuš, E. Scrocco, J. Tomasi: "Electrostatic interaction of a solute with a continuum. A direct utilizaion of AB initio molecular potentials for the prevision of solvent effects", *Chemical Physics* **1981**, *55*, 1, 117–129, DOI 10.1016/0301-0104(81)85090-2.

[8] S. Miertus, J. Tomasi: "Approximate evaluations of the electrostatic free energy and internal energy changes in solution processes", *Chemical Physics* **1981**, *65*, 239–245, DOI 10.1016/0301-0104(82)85072-6.

[9] S. Karbalaei Khani, A. Marefat Khah, C. Hättig: "COSMO-RI-ADC(2) excitation energies and excited state gradients", *Phys. Chem. Chem. Phys.* **2018**, *20*, 16354–16363, DOI 10.1039/C8CP00643A.

[10] Y. J. Franzke, C. Holzer, J. H. Andersen, T. Begušić, F. Bruder, et al.: "TURBOMOLE: Today and Tomorrow", *J. Chem. Theory Comput.* **2023**, *19*, 20, 6859–6890, DOI 10.1021/acs.jctc.3c00347.

[11] M. J. Frisch, G. Trucks, H. Schlegel, G. E. Scuseria, M. A. Robb, et al., Gaussian 16, Revision B.01, **2016**.

[12] F. Neese: "The ORCA program system", *WIREs Comput Mol Sci* **2012**, *2*, 1, 73–78, DOI 10.1002/wcms.81.

[13] F. Neese: "Software update: The ORCA program system—Version 5.0", *WIREs Comput Mol Sci* **2022**, *12*, 5, e1606, DOI 10.1002/wcms.1606.

[14] A. D. Becke: "Density-functional thermochemistry. III. The role of exact exchange", *The Journal of Chemical Physics* **1993**, *98*, 7, 5648–5652, DOI 10.1063/1.464913.

[15] A. McLean, G. Chandler: "Contracted Gaussian basis sets for molecular calculations. I. Second row atoms, Z= 11–18", *The Journal of chemical physics* **1980**, *72*, 10, 5639–5648, DOI 10.1063/1.438980.

[16] R. Krishnan, J. S. Binkley, R. Seeger, J. A. Pople: "Self-consistent molecular orbital methods. XX. A basis set for correlated wave functions", *The Journal of chemical physics* **1980**, *72*, 1, 650–654, DOI 10.1063/1.438955.

[17] M. M. Francl, W. J. Pietro, W. J. Hehre, J. S. Binkley, M. S. Gordon, D. J. DeFrees, J. A. Pople: "Self-consistent molecular orbital methods. XXIII. A polarization-type basis set for second-row elements", *The Journal of Chemical Physics* **1982**, *77*, 7, 3654–3665, DOI 10.1063/1.444267.

[18] F. Weigend, R. Ahlrichs: "Balanced basis sets of split valence, triple zeta valence and quadruple zeta valence quality for H to Rn: Design and assessment of accuracy", *Physical Chemistry Chemical Physics* **2005**, *7*, 18, 3297–3305, DOI 10.1039/B508541A.

[19] J. Širůček, B. Le Guennic, Y. Damour, P.-F. Loos, D. Jacquemin: "Excited-State Absorption: Reference Oscillator Strengths, Wave Function, and TDDFT Benchmarks", *Journal of Chemical Theory and Computation* **2025**, *21*, 9, 4688–4703, DOI 10.1021/acs.jctc.5c00159.

[20] L. Köttner, E. Ciekalski, H. Dube: "Peri-Anthracenethioindigo: A Scaffold for Efficient All-Red-Light and Near-Infrared Molecular Photoswitching", *Angewandte Chemie* **2023**, *135*, 52, e202312955, DOI 10.1002/ange.202312955.

[21] J. J. Snellenburg, S. Laptenok, R. Seger, K. M. Mullen, I. H. van Stokkum: "Glotaran: A Java-based graphical user interface for the R package TIMP", *Journal of Statistical Software* **2012**, *49*, 1–22, DOI 10.18637/jss.v049.i03.

[22] S. Grimme, S. Ehrlich, L. Goerigk: "Effect of the damping function in dispersion corrected density functional theory", *Journal of Computational Chemistry* **2011**, *32*, 1456–1465, DOI 10.1002/jcc.21759.
